# Dual Brachial Plexus Block for Distal Biceps Repair: A Case Report

**DOI:** 10.7759/cureus.94063

**Published:** 2025-10-07

**Authors:** Hannah R Popper, Joseph Massaglia, Armen Voskeridjian, Michael Rivlin

**Affiliations:** 1 Orthopaedic Surgery, Jefferson Health New Jersey, Philadelphia, USA; 2 Anesthesiology, United Anesthesia Services, PC, Philadelphia, USA; 3 Division of Hand Surgery, Rothman Orthopaedic Institute, Philadelphia, USA

**Keywords:** brachial plexus block, distal biceps repair, orthopedic hand surgery, orthopedic surgery, regional anesthesia, upper extremity

## Abstract

A 35-year-old male presented with a right distal biceps tear. The patient was scheduled for a distal biceps repair at an ambulatory surgical center. Given the patient’s body mass index of 56.68 kg/m^2^, the surgery center’s anesthesiologist recommended the use of a dual brachial plexus regional nerve block to avoid general anesthesia. While the use of dual brachial plexus blocks has been shown to be successful for shoulder procedures, their use in the distal humerus and elbow regions has not been fully established. Under ultrasound guidance, a combination of interscalene and supraclavicular nerve blocks was used as the sole anesthesia in addition to the use of midazolam for light sedation for the length of the procedure. The use of the dual block provided adequate biceps relaxation and anesthesia for a successful distal biceps repair using suture anchor repair. Dual regional blocks may be considered for patients, especially those with multiple comorbidities for whom general anesthesia poses an increased risk of complications, such as deep vein thrombosis, pulmonary embolism, and intraoperative bleeding.

## Introduction

Upper extremity surgery can be performed under general, regional, or local anesthesia or a combination of these modalities. Despite the widespread use of general anesthesia in hand and upper limb operations, regional anesthesia offers several advantages. Compared to general endotracheal anesthesia, regional blockade decreases intraoperative and postoperative analgesic requirements, improves hemodynamic stability, enhances operating room efficiency, reduces length of stay in the post-anesthesia care unit, and decreases unplanned admissions for pain control [1]. In addition, ultrasound guidance demonstrated excellent safety, with no pulmonary or neurovascular complications [2]. Due to these benefits, regional anesthesia has been a preferred technique in orthopedic procedures [3,4].

Regional anesthesia can be divided into two main categories, namely, neuraxial and peripheral nerve blocks [4]. Neuraxial anesthesia involves needle or catheter placement into the epidural or subarachnoid space (as in epidural or spinal anesthesia) [3,4]. Peripheral nerve blocks, such as those used in this case, involve injecting a local anesthetic near a specific nerve or nerve bundle [4].

The brachial plexus block is an effective regional anesthetic technique that can provide motor and sensory blockade of the upper extremity. It can be performed at various levels of the brachial plexus, i.e., interscalene, supraclavicular, infraclavicular, and axillary. Choosing the appropriate level depends on the surgical site and procedure complexity. The interscalene block, the most performed, is ideal for shoulder surgery but provides incomplete blockade around the elbow due to its distance from the inferior trunk [5]. The supraclavicular block targets all branches of the brachial plexus due to the compact arrangement of the trunks at this level, but without supplemental injections, it should not be used for upper arm surgery, given its poor sensory blockade of the shoulder girdle region [6]. Achieving complete surgical anesthesia for the entire upper extremity with a single block is often challenging, and there is limited recent literature presenting cases of dual brachial plexus anesthesia for upper extremity surgery, especially that of the lower arm, including the use for procedures such as distal biceps repair.

We present a case involving an adult male who underwent distal biceps repair using a dual brachial plexus block at the interscalene and supraclavicular levels as the sole anesthetic. The interscalene block managed tourniquet pain, while the supraclavicular block anesthetized the surgical field. This technique provided effective motor and sensory blockade, allowing for the avoidance of general anesthesia in a high-risk patient.

## Case presentation

The patient was a 35-year-old, right-hand-dominant Caucasian male with a body mass index (BMI) of 56.68 kg/m^2^ (5'3", 320 lbs) and a past medical history of depression, hypertension, and obesity. The patient was scheduled for a right distal biceps repair at an ambulatory surgical center. He sustained the injury while lifting a box of chlorine in his role as a pool company manager. He heard a “pop” and subsequently experienced soreness, bruising, and decreased elbow range of motion. Upon presentation, he had bruising at the elbow and tenderness over the bicipital tubercle. A positive Hook test and ultrasound confirmed a distal biceps rupture. No fractures or dislocations were noted on X-ray of the right elbow.

Given the patient’s age, hand dominance, and activity level, he was indicated for a distal biceps repair. For patients with a BMI >40 kg/m^2^, anesthetic decisions at our facility are made on a case-by-case basis by the anesthesiology team. There is no fixed BMI cutoff; the anesthesiologist evaluates airway anatomy and weight distribution individually. Although the surgeon requested total intravenous anesthesia with regional block, the anesthesiologist advised against it due to the patient’s comorbidities, most notably due to the patient’s BMI.

Preoperatively, the patient’s weight distribution and airway were evaluated. These were both found to be acceptable for surgery at the ambulatory surgical center. The Mallampati score was 2, with good mouth opening, >3 fingerbreadths of thyromental distance, and excellent neck extension [7]. The anesthesiologist recommended a dual brachial plexus nerve block with light sedation and advised against using propofol, making this a true light sedation-only case.

This was extensively discussed with the patient preoperatively, emphasizing the benefits of this approach while explaining the risks. The patient understood and agreed to both brachial plexus blocks and sedation for the case. The patient was assured that he would not feel pain, but may feel manipulation of the tissue throughout the procedure, such as pulling or tugging. We discussed that the patient would likely sleep through the procedure, but waking up should not be a cause for concern or anxiety. If this occurred, the patient was instructed to alert the anesthesiologist that he would like to be made “sleepier” and more medication would be given.

**Figure 1:**
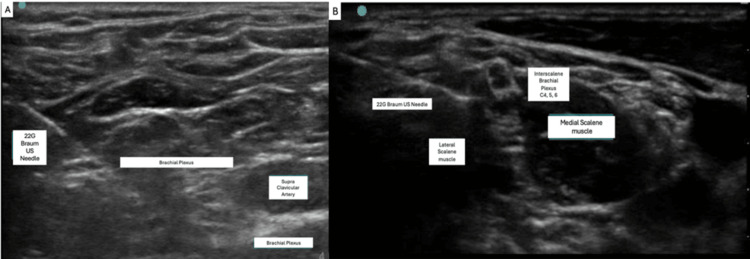
Ultrasound images of supraclavicular and intrascalene level. Ultrasound images of the supraclavicular (A) and the interscalene level (B), which were used to guide ropivacaine injection during the case.

The anesthesiologist used a dual block technique as described in prior studies under ultrasound guidance [8]: 7 mL of 0.5% ropivacaine at the level of the interscalene muscles and 23 mL of 0.5% ropivacaine by the supraclavicular artery (Figure 1). Dexamethasone (4 mg) was added to prolong block duration from an average of 12 hours (9-15 hours) to approximately 24 hours (18-30 hours). The nerve blocks were administered without incident with 2 mg of midazolam and 100 µg of fentanyl for initial sedation. In the operating room, the patient received an additional 4 mg of midazolam in two doses of 2 mg each. The total time in the operating room was 1 hour and 50 minutes.

The dual block provided excellent arm anesthesia and biceps relaxation, facilitating surgical manipulation without concerns of the patient’s vital signs or airway compromise. The distal bicep tendon was found to be retracted as expected, and the block allowed adequate muscle relaxation for successful retrieval and suture repair back to the bicipital tuberosity of the radius using two anchors. The surgical team noted no difference compared to traditional anesthesia methods. Excellent excursion, as well as biceps tension after repair, was achieved intraoperatively. After repair, appropriate hemostasis was achieved with no complications, and the patient was transferred to the recovery area in stable condition. Immediately postoperatively, the patient was placed in a long arm splint and was instructed to remain non-weight-bearing. At two weeks postoperatively, the patient was transitioned to an elbow brace, limiting range of motion from 30 to 90 degrees with a half-pound weight restriction. At six weeks postoperatively, the patient was instructed to complete a course of physical therapy for gradual return to strengthening and full activities over the course of four weeks. At 10 weeks postoperatively, the patient was cleared for return to work at full duty and had no complications.

## Discussion

Morbid obesity poses a significant challenge in ambulatory upper extremity surgery. Obesity is associated with airway-related comorbidities such as asthma and obstructive sleep apnea, increasing anesthesia risks [9]. While obese patients may experience higher block failure rates, studies show that regional anesthesia provides similar postoperative pain control, unanticipated admissions, and overall satisfaction in ambulatory settings [10].

Achieving complete upper extremity blockade with a single block is difficult. Dual blocks may reduce the need for general anesthesia and minimize anesthesia-related complications, particularly airway difficulties. Interscalene and supraclavicular blocks are established for shoulder surgery [11], but optimal anesthesia for the distal humerus and elbow remains debated.

Dhir et al. [12] demonstrated that the supraclavicular and infraclavicular blocks were equally effective for ambulatory elbow surgery with similar block onset times and failure rates. They found no difference in the rate of conversion to general anesthesia or postoperative pain scores in the immediate postoperative period. Other studies argue for axillary blocks due to better sensory coverage and lower complication rates [13,14]. Schroeder et al. [13] showed axillary nerve blocks provided superior anesthesia compared to supraclavicular and infraclavicular nerve blocks for elbow procedures, with an 86% success rate and no respiratory compromise.

Literature on combined interscalene and supraclavicular blocks for distal humerus and elbow procedures is limited. This dual block technique was performed for intramedullary nailing of a humerus with multiple pathologic fractures in a patient with end-stage liver disease and hepatocellular carcinoma with metastasis to the lung. The anesthesiologist was able to avoid intubation, which was very important given this patient’s severely compromised respiratory function. In addition, by avoiding airway manipulation, the patient did not have to go to the hospital setting, which was advantageous during the SARS-CoV-2 pandemic [15].

Phrenic nerve palsy and subsequent diaphragmatic paresis are well-known complications of the interscalene block, with some studies showing that this occurs in almost every case [16]. To mitigate this risk, we reduced the interscalene volume to 7 mL, keeping the injection lateral to the brachial plexus. The second block allowed for reduced interscalene volume. Short-acting agents such as 2% mepivacaine could be used to minimize phrenic nerve impact when necessary. Although risk is low, there are also systemic risks of brachial plexus blocks, including effects of local anesthetic toxicity such as visual disturbances, agitation, dizziness, or muscle fibrillations [6].

This report is one instance in which this dual brachial plexus block was successful for use in the distal humerus and elbow area. This report demonstrates the effective use of a dual brachial plexus block in a procedure lasting under two hours. However, this case alone cannot provide adequate evidence for widespread use. Larger case series are needed to provide greater evidence of the use of this technique for more distal upper extremity procedures, as well as use in procedures that are two to three hours long, to establish broader utility in upper extremity surgery. In addition, patients should be assessed postoperatively to assess for any complications or postoperative pain control assessments under this technique.

## Conclusions

This case highlights the use of dual brachial plexus nerve blocks as a sole anesthetic technique in a high-risk, morbidly obese patient undergoing ambulatory distal biceps repair. A single block would not have provided adequate anesthesia, particularly with tourniquet use. We present this case to describe the utility of a dual brachial plexus nerve block to provide effective anesthesia to the entire upper extremity safely, a technique less commonly demonstrated in the current literature. This is useful for patients with comorbid conditions precluding other anesthesia options. Further studies are warranted to assess broader use in upper extremity surgery.
